# Novel Approach for Assessing Postinfarct Myocardial Injury and Inflammation Using Hybrid Somatostatin Receptor Positron Emission Tomography/Magnetic Resonance Imaging

**DOI:** 10.1161/CIRCIMAGING.122.014538

**Published:** 2023-01-17

**Authors:** Andrej Ćorović, Deepa Gopalan, Christopher Wall, Marta Peverelli, Stephen P. Hoole, Patrick A. Calvert, Roido Manavaki, Tim D. Fryer, Luigi Aloj, Martin J. Graves, Martin R. Bennett, James H.F. Rudd, Jason M. Tarkin

**Affiliations:** 1 Section of CardioRespiratory Medicine (A.C., C.W., M.P., S.P.H., P.A.C., M.R.B., J.H.F.R., J.M.T.), University of Cambridge, United Kingdom.; 2 Department of Radiology (R.M., L.A., M.J.G.), University of Cambridge, United Kingdom.; 3 Department of Clinical Neurosciences (T.D.F.), University of Cambridge, United Kingdom.; 4 Department of Radiology, Cambridge University Hospitals NHS Trust, United Kingdom (D.G.).; 5 Department of Cardiology, Royal Papworth Hospital NHS Trust, United Kingdom (S.P.H., P.A.C.).

**Keywords:** inflammation, magnetic resonance imaging, myocardial infarction, positron emission tomography, receptors, somatostatin

Postinfarct inflammation and its resolution modulate ischemic injury after myocardial infarction (MI). While cardiac magnetic resonance imaging (MRI) is useful for assessing ventricular function, viability, and structural complications after MI, as well as detecting edema associated with acute inflammation, it lacks specificity for immune cell activity and may be less sensitive for identifying persistent, low-grade inflammation. Positron emission tomography (PET) imaging with ^18^F-Fluorodeoxyglucose (FDG) and other more specifically targeted tracers could have a role for quantifying infarct-related inflammation and identifying a link with adverse myocardial remodeling.^1^ Focal myocardial uptake of the SST_2_ (somatostatin receptor 2) PET tracer ^68^Ga-DOTATATE has been observed in patients with prior infarction^2^; however, the clinical relevance of this finding remains unknown. Here, we report an illustrative case from an ongoing study aimed to evaluate a novel, integrated approach for assessing postinfarct myocardial injury and inflammation using hybrid SST_2_ PET/MRI.

A 36-year-old man with no prior medical history or cardiovascular risk factors presented with severe, sudden-onset chest pain. Cardiorespiratory examination was normal. The ECG showed sinus rhythm with evolving inferior ST-segment elevation without reciprocal changes. He underwent primary percutaneous coronary intervention to an occluded obtuse marginal branch of the left circumflex artery (Figure 1A and 1B). Reperfusion was achieved within 3 hours of symptom onset. Medical therapy with dual antiplatelet agents, a statin, beta-blocker, and angiotensin-converting enzyme inhibitor was initiated.

**Figure 1.**
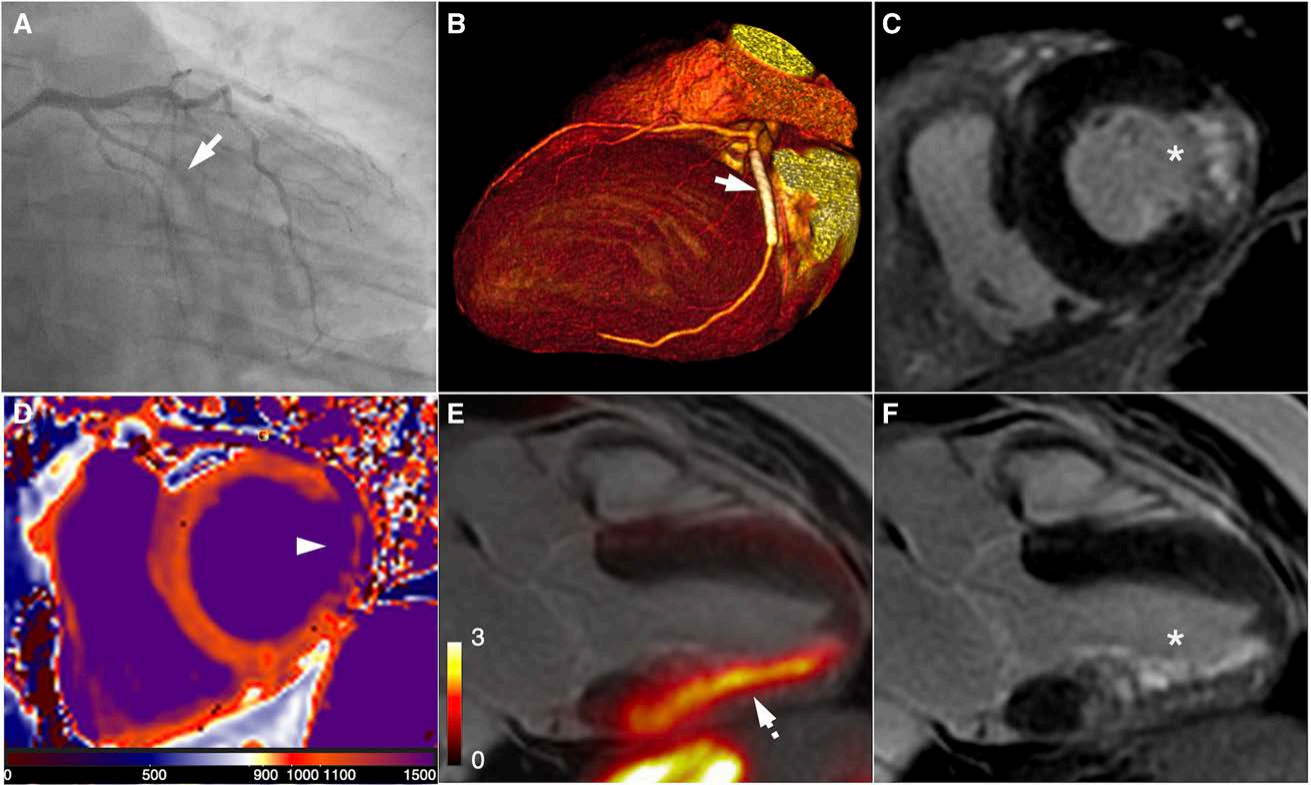
**Baseline ^68^Ga-DOTATATE positron emission tomography (PET)/magnetic resonance imaging (MRI) after myocardial infarction. A**, Coronary angiography showing an occluded obtuse marginal branch of the left circumflex artery (arrow) at the time of initial myocardial infarction (MI) presentation; (**B**) 3-dimensional volume-rendered computed tomography coronary angiography after percutaneous coronary stenting (arrow). PET/MRI performed on day 11 post-infarct: (**C**, short-axis view; **F**, 3-chamber view) late gadolinium enhancement (LGE) MRI confirms areas of near full thickness basal infero-lateral MI (asterisk) and sub-endocardial mid to apical inferolateral infarction; (**D**) T1-mapping in basal short-axis view corroborating the extent of infarct (arrowhead); (**E**) increased ^68^Ga-DOTATATE PET uptake (dashed arrow) co-localizes with the area of infarct.

Initial blood tests showed a total cholesterol level of 5.4 mmol/L, with triglycerides of 2.49 mmol/L, and an LDL of 3.39 mmol/L. The total white cell count was elevated at 18.4x 10^9^/L, with a neutrophilia. The C-reactive protein level was normal at <3 mg/L and the peak hsTnI (high-sensitivity Troponin I) concentration was >25 000 ng/L (normal <58 ng/L). Echocardiography demonstrated normal left ventricular function overall with a biplane ejection fraction of 58%, akinesis of the basal-to-mid inferolateral wall, and hypokinesis of the basal-to-mid anterolateral wall.

The patient was enrolled in a multi-modality cardiac imaging research study (REGISTRATION: URL: https://www.clinicaltrials.gov; Unique identifier: NCT04073810), involving serial ^68^Ga-DOTATATE PET/MRI, cardiac MRI and CT coronary angiography with 2-year follow-up. Baseline PET/MRI was performed using a hybrid PET/MRI scanner (SIGNA, GE Healthcare) on day 11 post-MI. Fifty minutes after an injection of 238 MBq ^68^Ga-DOTATATE, PET images were acquired for 35-minutes in a cardiac bed position. Simultaneously acquired 3T MRI included 3-plane breath-held proton weighted, blood-suppressed single-shot fast-spin echo, 2D steady-state free precession cine imaging of the ventricles, T2-weighted edema imaging, T1 and T2 mapping, and late gadolinium enhancement. Static PET images were reconstructed from list mode data using iterative time-of-flight (256×256 matrix, Q.Clear b=350), and a free-breathing 2-point DIXON MR imaging sequence for attenuation correction.

Baseline MRI showed preserved left ventricular function, with persistent regional wall abnormalities (supplemental material: short-axis cine MRI acquired shortly after gadolinium contrast, Video S1). There was increased signal on T2-weighted imaging in the basal anterolateral, mid inferolateral, and apical lateral walls signifying edema, with corresponding areas of near full-thickness and sub-endocardial late gadolinium enhancement (Figures 1C, 1F, and 2A). ^68^Ga-DOTATATE PET demonstrated increased uptake (Figures 1E and 2G) in the infarct zone defined by late gadolinium enhancement and T1 mapping (Figure 1D). The maximum Standardised Uptake Values (SUV_max_) and Tissue-to-Background Ratios (TBR_max_) normalized for blood-pool activity were 2.8 and 8.0, respectively, in the infarct versus 1.3 and 3.6 in the remote myocardium.

**Figure 2.**
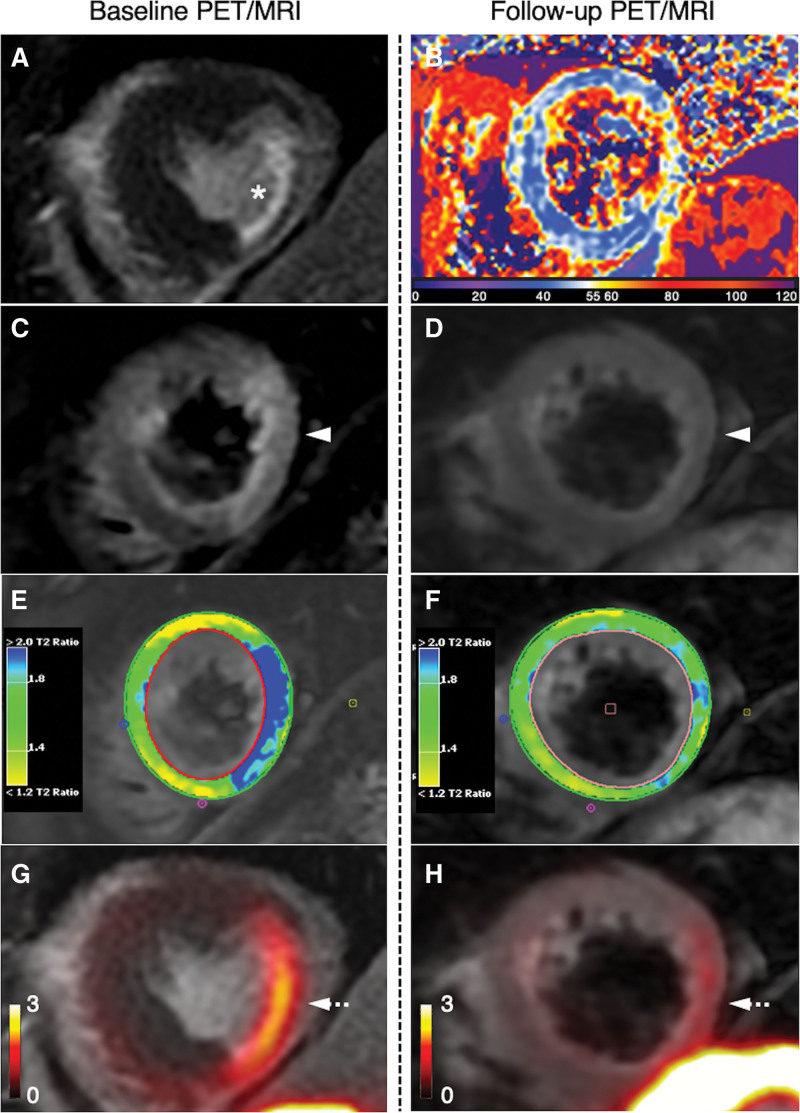
**Comparison of baseline and 3-month follow-up ^68^Ga-DOTATATE positron emission tomography (PET)/magnetic resonance imaging (MRI).** Baseline PET/MRI: (**A**) Late gadolinium enhancement (LGE) MRI showing sub-endocardial myocardial infarction (asterisk) in the mid inferolateral segment, with corresponding (**C**) high signal (arrowhead) on T2-weighted imaging; and (**G**) increased ^68^Ga-DOTATATE PET uptake (dashed arrow). **E**, Color-map demonstrates myocardium-to-muscle T2 ratio. Follow-up PET/MRI performed 102 days postinfarct: (**B**) T2-mapping; (**D**) resolution of edema on T2-weighted imaging; (**F**) myocardium-to-muscle T2 ratio; (**H**) residual infarct-related ^68^Ga-DOTATATE signal (dashed arrow). LGE imaging was not repeated at follow-up.

Over the following 3 months, the patient remained well. Follow-up PET/MRI performed 102 days post-MI using the same scanner and imaging protocol with an injected activity of 242 MBq showed a reduction, but not resolution, of the ^68^Ga-DOTATATE PET signal (infarct SUV_max_ 2.0; infarct TBR_max_ 5.5; Figure 2H). Although there was no residual edema visible on T2-weighted MRI at follow-up (Figure 2D and 2F) compared with baseline (Figure 2C and 2E), quantitative T2 values were higher in the infarcted myocardium than remote region (infarct: 62 ms; remote: 51 ms; average noninfarct T2 value for scanner <53 ms; Figure 2B). In contrast to the abnormal PET and MRI findings consistent with ongoing myocardial inflammation, blood tests at day 102 showed normalization of hsTnI (15 ng/L from 206 ng/L) and reduction in NTproBNP (122 pg/mL from 362 pg/mL) levels, compared with day 11 values. High-sensitivity CRP remained low (0.27 mg/L from 0.69 mg/L).

SST_2_ PET imaging holds major promise as a novel marker of cardiovascular inflammation across several disease entities. *SSTR2* gene expression is upregulated by macrophages stimulated in vitro, and the SST_2_ receptor is co-expressed by CD68-positive macrophages in carotid endarterectomy specimens from stroke patients,^1^ as well as endomyocardial biopsies from patients with cardiac sarcoidosis and myocarditis.^3^ Unlike ^18^F-FDG, somatostatin receptor PET tracers such as ^68^Ga-DOTATATE have very low background activity in the healthy heart allowing for unhindered assessment of pathological myocardial inflammation without the need for dietary myocardial suppression. Indeed, clear infarct-related ^68^Ga-DOTATATE PET/CT uptake was observed in patients with both recent MI and chronic ischemic left ventricular dysfunction, in a post hoc analysis of the VISION study.^2^ The mechanism of ^64^Cu-DOTATATE binding to SST_2_ receptors expressed by inflammatory macrophages within recently infarcted myocardial tissue has been shown in a mouse model, using a combination of in vivo PET/CT imaging and ex vivo radiometric and immunologic assays.^4^ In that study, ^64^Cu-DOTATATE uptake within cell-sorted macrophages from infarcted mouse myocardium was 3-fold higher than ^18^F-FDG.

This imaging case report highlights a newly emerging molecular imaging method for interrogating specific components of the immune response to ischemic myocardial damage. In this instance, ^68^Ga-DOTATATE PET uptake was closely colocalized with MRI features of recent MI, and later revealed residual inflammation that was not overtly visible on MRI, nor detected by blood biomarkers. Research is ongoing to confirm the cellular origins of post-MI ^68^Ga-DOTATATE PET signal within inflamed and infarcted human myocardial tissue, and to test its association with longer-term ischemic myocardial remodeling. In the future, this approach could be clinically useful for informing the design and use of advanced immunomodulatory therapies for patients with chronic ischemic cardiomyopathy. Simultaneous PET/MRI acquisition presents a unique opportunity for a truly comprehensive assessment of cardiac function, tissue characterization, and viability from MRI, alongside the superior sensitivity and inflammatory cell-specificity afforded by molecular imaging.

## Article Information

### Sources of Funding

This work was funded by grants to Dr Tarkin from the Wellcome Trust (Clinical Research Career Development Fellowship, 211100/Z/18/Z); and the British Heart Foundation (Clinical Research Training Fellowship for Dr Ćorović, FS/CRTF/20/24035).This work was additionally supported by the Cambridge BHF Centre of Research Excellence (18/1/34212); the NIHR Cambridge Biomedical Research Centre; the Cancer Research UK Cambridge Centre (A25177); and the Wolfson Brain Imaging positron emission tomography/magnetic resonance imaging team.

### Disclosures

None.

### Supplemental Material

Video S1

## Supplementary Material
