# Supplementary material for: Novel Approach for Assessing Postinfarct Myocardial Injury and Inflammation Using Hybrid Somatostatin Receptor Positron Emission Tomography/Magnetic Resonance Imaging
Source: Circ Cardiovasc Imaging. 2023 Jan 17;16(1):e014538. doi: 10.1161/CIRCIMAGING.122.014538 (PMC9848209; doi:10.1161/CIRCIMAGING.122.014538)
Supplement: Supplementary file 1 [file hci-16-e014538-s001.pdf]

**SUPPLEMENTAL MATERIAL**

**Video Legend:**

**Video S1.** Short-axis steady state free precession cine magnetic resonance imaging.
